# Emerging Indications for Interventional Oncology: Expert Discussion on New Locoregional Treatments

**DOI:** 10.3390/cancers15010308

**Published:** 2023-01-02

**Authors:** Roberto Iezzi, Afshin Gangi, Alessandro Posa, Uei Pua, Ping Liang, Ernesto Santos, Anil N. Kurup, Alessandro Tanzilli, Lorenzo Tenore, Davide De Leoni, Dimitrios Filippiadis, Felice Giuliante, Vincenzo Valentini, Antonio Gasbarrini, Shraga N. Goldberg, Martijn Meijerink, Riccardo Manfredi, Alexis Kelekis, Cesare Colosimo, David C. Madoff

**Affiliations:** 1 Department of Diagnostic Imaging, Oncologic Radiotherapy and Hematology, Fondazione Policlinico Universitario A. Gemelli IRCCS, L.go A. Gemelli 8, 00168 Rome, Italy; 2 Università Cattolica del Sacro Cuore di Roma, Largo Francesco Vito 1, 00168 Rome, Italy; 3 Department of Interventional Radiology, University Hospital of Strasbourg, 67091 Strasbourg, France; 4 Department of Diagnostic Radiology, Tan Tock Seng Hospital, Singapore 308433, Singapore; 5 Department of Interventional Ultrasound, PLA Medical College & Fifth Medical Center of Chinese PLA General Hospital, Beijing 100853, China; 6 Department of Radiology, Interventional Radiology Service, Memorial Sloan Kettering Cancer Center, New York, NY 10065, USA; 7 Department of Radiology, Mayo Clinic, 200 1st St. SW, Rochester, MN 55905, USA; 8 2nd Department of Radiology, University General Hospital “ATTIKON” Medical School, National and Kapodistrian University of Athens, 1 Rimini Str., 12462 Athens, Greece; 9 Hepatobiliary Surgery Unit, Fondazione Policlinico Universitario A. Gemelli IRCCS, L.go A. Gemelli 8, 00168 Rome, Italy; 10 Internal Medicine and Gastroenterology Unit, Fondazione Policlinico Universitario A. Gemelli IRCCS, L.go A. Gemelli 8, 00168 Rome, Italy; 11 Division of Image-Guided Therapy, Department of Radiology, Hadassah Hebrew University Medical Center, Jerusalem 12000, Israel; 12 Department of Radiology and Nuclear Medicine, Amsterdam University Medical Centers, De Boelelaan 1117, 1081HV Amsterdam, The Netherlands; 13 Department of Radiology and Biomedical Imaging, Section of Interventional Radiology, Yale School of Medicine, 330 Cedar St., TE-2, New Haven, CT 06510, USA

**Keywords:** interventional oncology, head and neck cancer, spinal tumors, breast cancer, cholangiocellular carcinoma, lymphatics, bone metastases, ablation, radioembolization, cementoplasty

## Abstract

**Simple Summary:**

Interventional oncology provides minimally invasive alternatives to many traditional medical and surgical treatments, offering a potential cure, control, or palliative care for many types of cancer patients. The aim of this article is to comprehensively review new indications for locoregional interventional oncology treatments and summarize the expert discussion and report from the “MIOLive Meets SIO” (Society of Interventional Oncology) session during the last Mediterranean Interventional Oncology Live congress held in Rome, Italy.

**Abstract:**

Interventional oncology (IO) employs image-guided techniques to perform minimally invasive procedures, providing lower-risk alternatives to many traditional medical and surgical therapies for cancer patients. Since its advent, due to rapidly evolving research development, its role has expanded to encompass the diagnosis and treatment of diseases across multiple body systems. In detail, interventional oncology is expanding its role across a wide spectrum of disease sites, offering a potential cure, control, or palliative care for many types of cancer patients. Due to its widespread use, a comprehensive review of the new indications for locoregional procedures is mandatory. This article summarizes the expert discussion and report from the “MIOLive Meet SIO” (Society of Interventional Oncology) session during the last MIOLive 2022 (Mediterranean Interventional Oncology Live) congress held in Rome, Italy, integrating evidence-reported literature and experience-based perceptions. The aim of this paper is to provide an updated review of the new techniques and devices available for innovative indications not only to residents and fellows but also to colleagues approaching locoregional treatments.

## 1. Introduction

Interventional oncology (IO) employs image-guided techniques to perform minimally invasive procedures, providing lower-risk alternatives to many traditional medical and surgical therapies in cancer patients. Since its advent, due to rapidly evolving research development, its role has expanded to encompass the diagnosis and treatment of diseases across multiple body systems. In detail, IO is expanding its role across a wide spectrum of disease sites, offering a potential cure, control, or palliative care for many types of cancer patients. Due to its widespread use, a comprehensive review of the new indications for locoregional procedures is mandatory [1]. This article summarizes the expert discussion and report from the MIOLive meets SIO (Society of Interventional Oncology) session during the last MIOLive 2022 (Mediterranean Interventional Oncology Live) congress held in Rome, Italy, integrating evidence-reported literature and experience-based perceptions. The aim of our paper is to integrate evidence-reported literature and experience-based perceptions while attempting to make the information easy to access in order to assist not only residents and fellows who are training in interventional radiology but also practicing colleagues who are attempting to gain further expertise with this locoregional procedures and to also highlight recent data and strategies to treat disease states not yet routine in IO practice.

## 2. Spine, Skull, and Skull Base Tumors

IO is one of the fastest growing clinical specialties for the treatment of skull and skull base tumors. CT and MRI are the preferred techniques to guide percutaneous interventions in these locations [2]. Representing a complex location, ablative procedures cannot be considered a “definitive” therapy yet. It is important to focus on the clinical picture of every single patient to choose the best therapy line in a multidisciplinary way.

### 2.1. Spinal Tumors

Spinal tumors represent one of the main targets of ablative procedures, particularly osteoid osteoma, osteoblastoma, and chondroblastoma as well as spine metastases. Technical progress in interventional radiology made the treatment of previously untreatable spinal tumors possible, such as those located in the body of cervical vertebrae. Among interventional radiology treatments in this district, radiofrequency ablation can be used to obtain local tumor control and pain relief alone or in combination with vertebroplasty procedures with good tumor control and low complication rates [3].

Cryoablation represents another safe and effective therapeutic technique [4]. A cryo-probe is positioned in the lesion whereas a thermocouple is placed in the posterior wall of the vertebral soma. It is important to remember that the freezing process continues for about 1 min after the cryo-probe is switched off; therefore, this process must be taken into account when calculating ablation times.

Thirty-minute neurophysiological monitoring, performed by placing electrodes in various districts of the body, can be helpful to monitor motor and sensitive systems, safely perform percutaneous ablations, and promptly stop the procedure if something is wrong. General anesthesia should be obtained before percutaneous ablation of these districts. When needed, hydrodissection is performed soon before ablation.

### 2.2. Desmoid Tumors

Cryoablation of large symptomatic cervical desmoid tumors that do not respond to first- and second-line medical treatment can be performed with multiple cryo-probes [5]. About 65% of desmoid tumors treated with cryoablation obtain good results.

### 2.3. Ear, Nose, and Throat (ENT) Tumors

ENT tumors can be represented by various histological entities (i.e., squamous cell carcinoma, adenocarcinoma, muco-epidermoid carcinoma, adenoid cystic carcinoma, and esthesioneuroblastoma). Cryoablation of these tumors is usually performed as a last-line treatment after surgery, radiation therapy, and chemotherapy in patients with no other treatment options [6]. The advantage of cryoablation is represented by the possibility of good ablation margins for better local disease control. Complications of cryoablation in these tumors can be represented by inhalation pneumopathy and dysphonia (due to injury of the recurrent nerve) in the case of nearby neoplasms.

### 2.4. Intrasinusal Frontal Hemangioma

Hemangiomas located in the frontal sinus can be painful [7]. CT-guided cryoablation can be performed in these tumors when surgery is not feasible. However, a combined approach in association with a surgical team can be performed: Surgical endoscopic checks and guidance for correct positioning of the cryo-probes can be helpful. In the case of aggressive hemangiomas, it is mandatory to perform cryoablation to preserve skin tissues as well as the frontal cerebral lobe. Adjacent soft tissue edema can occur in the early postprocedural setting, which is usually self-limiting. MRI is the best technique in guiding cryoablation in the case of soft tissue tumors, as it can better show the lesion, probe as well as ice ball.

### 2.5. Palatine Nasopharyngeal Carcinoma

Cryoablation of deep nasopharyngeal tumors, such as posterior palatine ones, is challenging due to the difficult location of the lesion and complex cryo-probe(s) positioning. The lips and tongue must be immobilized, granting full access to the tumor. Cryoablation can be considered in the case of tumors that are not suitable for surgical resection.

### 2.6. Glioblastoma

Recurrent glioblastoma can be also treated with cryoablation [8]. The association of cryoablation and systemic therapies (chemotherapy or immunotherapy) or radiation therapy leads to better survival rates than those techniques alone. Cryoablation determines tumor necrosis while increasing the immune response, which can be useful for combined treatments. To reduce cerebral edema, necrotic postablative tumor tissue could be surgically removed.

## 3. Deep Spaces of the Neck

The literature on this topic is scarce with limited case series. Treatment planning is crucial, as the correct approach to tumors in this area is mandatory to achieve good and safe results. The main indication for ablation of neck lesions is a localized disease without any other local treatment options (e.g., unfeasible or exhausted surgery and/or radiotherapy) and only undergoing systemic therapy [9]. While ablation may have a limited impact on survival, it can have a positive impact on the quality of life through local tumor control, leading to pain relief, reduction of mass effect as well as delay in the usage of feeding tubes (i.e., percutaneous gastrostomy or jejunostomy) and/or tracheostomy due to tumor obstruction [10,11]. Of note, a complete ablation margin may not always be feasible due to the lesion’s dimension and location next to critical structures [12]. Approaches to lesions in deep spaces of the neck are retromaxillary, retromolar trigone, transglandular, and transmuscular flap. With those approaches, up to 95% of lesions can be reached and treated percutaneously (as opposed to transorally). Ablative treatments and cryoablation in particular can potentially fill a treatment gap in recurrent or nonsurgical head and neck tumors. It is technically feasible, but more data are required to clearly determine the case selection and outcome matrix.

**Figure 1:**
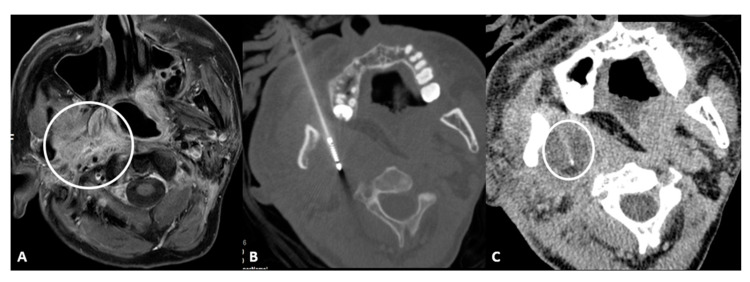
56-year-old male with neuropathic pain due to right oropharyngeal squamocellular carcinoma recurrence after tracheostomy, neck lymph node dissection, mandibulotomy, right oropharyngeal resection with free anterolateral thigh flap. (**A**) T1-weighted postcontrast axial MRI scan showing malignant involvement of trigeminal nerve (circle). (**B**) Axial CT scan showing cryoablation needle placement. (**C**) Intraprocedural axial CT scan showing ice ball formation around the needle (circle).

**Figure 2:**
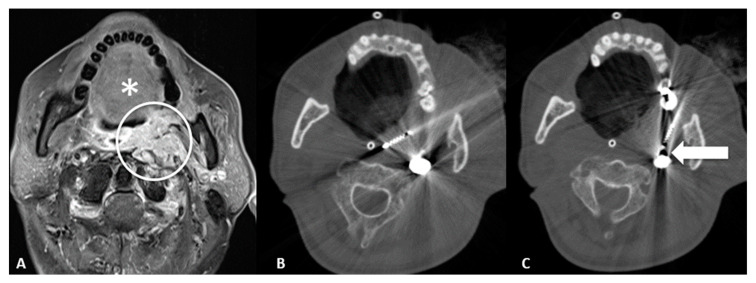
65-year-old male with trismus, gagging, airway compromise, and bleeding episodes due to recurrent nasopharyngeal carcinoma of the left retropharyngeal/paravertebral space with circumferential carotid artery involvement, after previous radiation therapy and left neck dissection. (**A**) T1-weighted postcontrast axial MRI scan showing malignant involvement of the left retropharyngeal space (circle). Notice the tongue (*) which can flop backward during the general anesthesia and could contact the ablation site; care must be taken in blocking the tongue prior to ablation. (**B**) Axial CT scan showing cryoablation needle placement through the retromolar trigone. (**C**) Axial CT scan showing the cryoablation needle near the previously embolized left carotid artery (arrow), which can lead to diving reflex.

**Figure 3:**
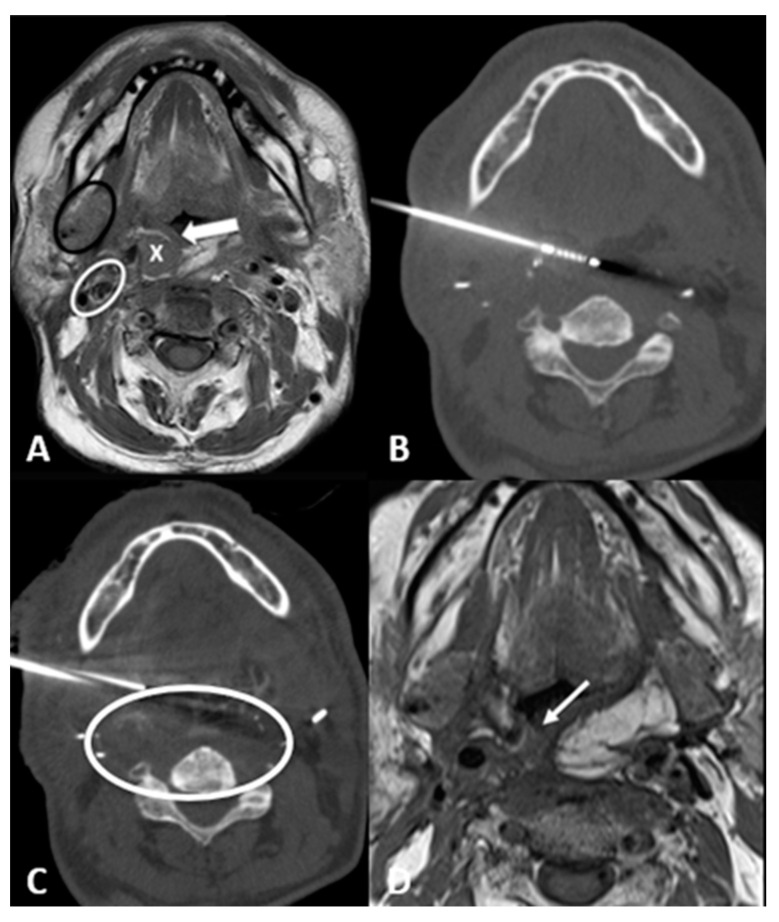
63-year-old male with recurrence of supraglottic squamocellular carcinoma which underwent previous total pharyngolaryngectomy and bilateral neck dissection. (**A**) T1-weighted unenhanced axial MRI scan showing the tumor recurrence (X), the mucosa of the neopharynx (arrow), the right submandibular gland (black circle), and the right carotid space (white circle). (**B**) Axial CT scan showing the cryoprobe positioned in the lesion through a trans-submandibular gland approach. (**C**) Axial CT scan showing parallel needle placement for hydrodissection of peritumoral tissue. (**D**) One-month T1-weighted unenhanced axial MRI scan showing the postablation tumor shrinkage (arrow).

**Figure 4:**
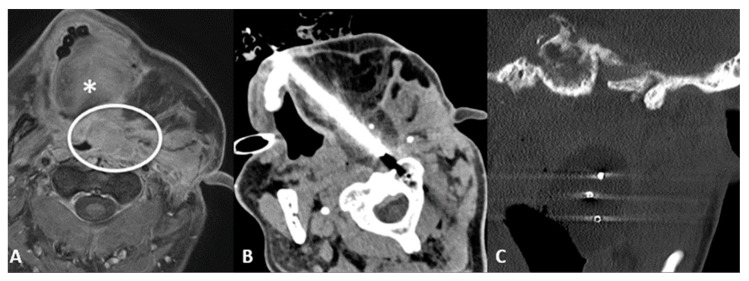
70-year-old male with airway impairment and left postauricular discharge. Previous nasopharyngeal carcinoma and radiation-induced squamous cell carcinoma of maxilla which underwent maxillectomy, mandibulectomy, parotidectomy, and myocutaneous flap reconstruction. (**A**) T1-weighted postcontrast axial MRI scan showing extensive tumor recurrence (white circle), posterior to the tongue (*), threatening the airways and invading the left external auditory canal. (**B**) Axial CT scan showing cryoablation needle placement through the myocutaneous flap. (**C**) Coronal-oblique multiplanar reconstruction CT scan showing the ice ball formed by the three cryoprobes.

- Retromaxillary: In the case of a retromaxillary approach, the maxilla is used as a landmark. This approach is mostly used for lesions located in the masticatory space and the pharyngeal and laryngeal mucosa (Figure 1).

- Retromolar trigone: This is the space between the last molar and the mandibular condyle. It is a space without many vessels/nerves or vital structures; therefore, it represents a safe approach to treating pharyngeal and laryngeal lesions. Tongue positioning is mandatory, as it can flop backward against the ablation site resulting in frost injury: Forceps and a laryngoscope should be used to displace the tongue with gauze away from the ablation site to avoid this occurrence. In the case of tumors near the carotid artery, the patient can manifest severe hypotension and bradycardia during ablation. The presence of coils or metallic devices in a previously embolized carotid artery leads both to a loss of the cold-sink effect as well as a stimulation of the vagus nerve with thermal conduction down the carotid body (leading to the diving reflex). Cessation of cryoablation usually leads to spontaneous resolution (Figure 2).

- *Transglandular*: This is a very useful approach in case of lesions in the pharyngeal space and prevertebral space, crossing the submandibular gland to reach these locations (Figure 3).

- Transflap: Useful in the case of early local tumor recurrence, in patients who underwent previous surgery, and myocutaneous flap reconstruction (Figure 4). The myocutaneous flap is usually devoid of significant vascularity and vital structures and presents a safe trajectory once it has been grafted [13].

## 4. Breast Cancer

Breast cancer (BC) is a common malignancy with the second mortality rate in women worldwide; in China, the 5-year survival rate of patients affected by BC is about 83% while in occidental countries, it can be up to 90% [14,15].

Surgical techniques (mastectomy) are usually associated with psychological disorders in about 70% of patients [16,17]. In the 1990s, breast-conserving operations were recommended, but the recurrence rate was up to 21.6%.

### 4.1. Locoregional Approaches

Common ablation techniques include cryoablation, laser, high-intensity focused ultrasound (HIFU), radiofrequency ablation (RFA), and microwave ablation (MWA) [18]. Ablation techniques are minimally invasive and do not leave any scars; the ablated lesions gradually shrink, becoming impalpable [19]. Ablation procedures can be repeated for new lesions so that it is possible to protect breast tissue to the largest extent. Ablations have a very low complication rate. Compared to other ablation modalities, MWA has the highest technical efficacy rates (90–95%) [20]. When compared to breast-conserving surgery, MWA obtained better results in edema drainage, operation time, estimated blood loss, and, especially, cosmetic results [21].

**Figure 5:**
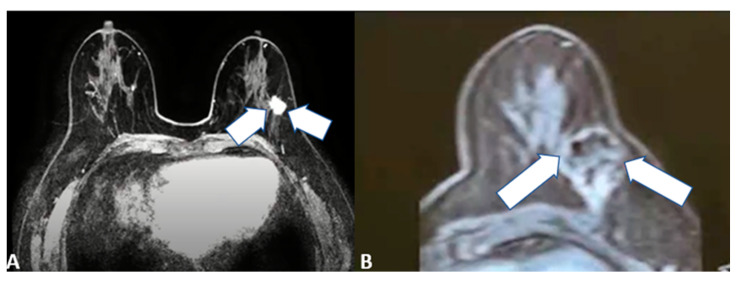
64-year-old female patient with left invasive T1 breast cancer of 11 mm with a molecular subtype Luminal-A. Contrast-enhanced T1-weighted MRI scan showing (**A**) baseline neoplastic lesion and (**B**) 5-month follow-up after microwave ablation with good ablation margins and central necrosis.

Target lesions should be smaller than 2 cm in size and located apart from the nipple (Figure 5) [22,23,24]. Histological examination should be negative for sentinel lymph nodes with no pathological axillary lymph nodes at imaging.

**Figure 6:**
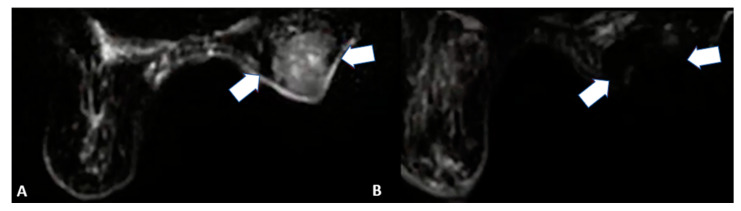
85-year-old female patient with invasive right breast cancer with axillary metastases. Diffusion-weighted imaging MRI scan showing (**A**) baseline large neoplastic lesion infiltrating the skin and soft tissues (arrows) and (**B**) 4-month follow-up after microwave ablation with absent signal restriction (arrows).

Ablative treatments can also represent a palliative ablation for larger lesions (Figure 6) as well as an alternative therapy for those patients who are not suitable for surgery or refuse resection and/or other treatment modalities (such as chemotherapy) for cosmetic reasons [25,26].

Contraindications of ablative techniques are severe dysfunction of blood coagulation, pregnancy, or breastfeeding and severe cardiac or pulmonary impairment [27]. Before ablation, a detailed ultrasound (US) scan should be executed to measure lesion size and location, to study lesion margins, blood supply, and adjacency, and to schedule the ablation protocol: puncture route, ablation power and time, and anesthesia method. A US-guided 16 G needle biopsy needs to be executed prior to ablation to confirm the pathological diagnosis and perform immuno-histochemical examinations.

### 4.2. Key Points

**Figure 7:**
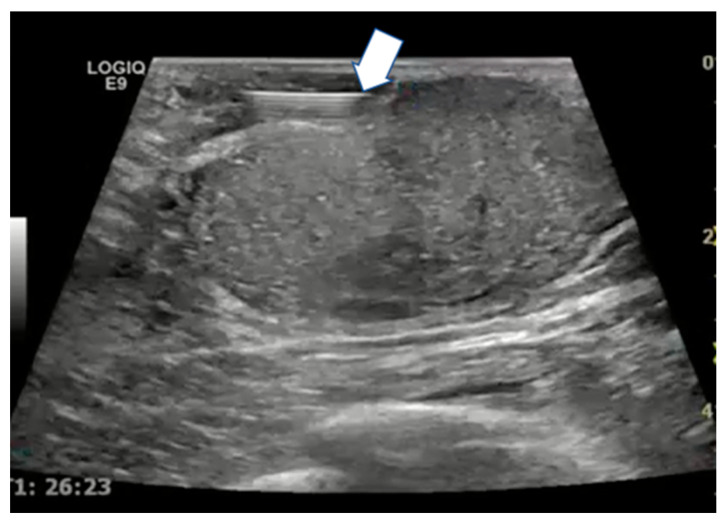
Ultrasound scan with linear probe showing the tip of the needle used for hydrodissection (arrow) to inject saline between the tumor and the skin or ductal structures.

If needed, hydrodissection can be performed by injecting saline solution between the tumor and the adjacent tissue using a 20/22 G needle to prevent thermal damage to nontarget structures (Figure 7) [24]. Different tumor histology needs different needle ablation power. For deeper tumors, 30–50 W of ablative power is recommended whereas for superficial ones, 20 W is the right ablation power due to the greater injury risk to the skin and soft tissues. Ablative approaches should be performed with a pullback technique to cover the tumor area [24].

## 5. Intrahepatic Cholangiocellular Carcinoma

Cholangiocellular carcinoma (CCA) is the second most common hepatic malignancy with an increasing incidence worldwide and poor prognosis: The overall survival is about 3 months, and the 5-year survival is less than 10% [28]. To date, the only curative treatment is surgical resection; however, less than 30% of patients are suitable for surgery [29]. Even with negative surgical margins, recurrence rates are very high (85% intrahepatic CCA—14% extrahepatic CCA) [30]. The median overall survival after resection is 18–33 months even with negative margins [31]. The CCA is very therapeutic resistant due to its marked genetic heterogeneity and complex tumor microenvironment [32].

### 5.1. Locoregional Approaches

The aim of locoregional therapies in intrahepatic CCA is to achieve oncological benefits, prevent tumor progression and its complication, improve overall survival, and represent a bridge to surgical resection or transplant [33]. Local ablative therapies are intended to eradicate all viable malignant cells within the designated target volume, sparing the normal surrounding tissues. These procedures can be repeatable and can also be done in an outpatient setting. Locoregional therapies also have an important role in the treatment of bleeding and pain management. At present, there are no formal guidelines for locoregional therapies in CCA management and unclear and limited literature evidence [34,35,36].

Standard indications for CCA ablation include ineligibility for surgery, small tumor size (up to 3 cm), and no extrahepatic spread. Lesion size is the most relevant factor to achieve complete ablation and reduced local recurrence rates or local tumor progression [33,37,38,39,40,41,42].

RFA is the most diffused locoregional treatment technique whereas MWA has some theoretical advantages (i.e., is less dependent on tissue’s electrical conductivity, is less influenced by the “heat-sink” effect, obtains higher intratumoral temperatures, reduced ablation time, and larger ablation zone) [43,44,45,46,47,48].

Many factors affect locoregional treatment outcomes: Intrahepatic CCA is a noncapsulated and tissue-infiltrating malignancy; therefore, a minimum of 10 mm of safety margin should be obtained to reduce the risk of local recurrence [49]. In addition, lymph node positivity and the superficial location of the tumor influence the results of the procedure. Due to the advanced stage of the diagnosis, most patients are not suitable for ablation; therefore, data on intrahepatic CCA ablation therapy are limited. In 2015, a review of seven observational studies on the ablation of primary and unresectable intrahepatic CCA was published: The overall survival ranged from 20 to 60 months, and treatment success depended on tumor size [41]. Three studies showed higher rates of residual neoplastic tissue when the tumor size was greater than 4.6 cm [50,51,52]. Another study showed a mean 5-year survival rate of 24% and a local tumor progression rate of 21% [53]. In conclusion, ablation prolongs survival rates in patients ineligible for surgery; furthermore, it is linked to lower complications, costs, and shorter hospital stays. A recent study comparing repeated resection versus thermal ablation in the treatment of tumor recurrence after the first resection showed that there were no differences in overall survival and disease-free survival rates [54]. Significant prognostic factors for overall survival included a solitary recurrent tumor and recurrent interval from the initial resection greater than 1 year. Major complications were more common in patients treated with repeated resections; therefore, it has been concluded that thermal ablation should be preferred over repeated resection in patients with intrahepatic recurrence smaller than 3 cm in size [54].

To date, transarterial therapies for intrahepatic CCA are indicated in cases of high tumor burden, in neoadjuvant settings, and when the position of the tumor is difficult/dangerous [55,56]. In a prospective trial with 115 patients who underwent conventional transarterial chemoembolization (TACE) with different combinations of mitomycin, gemcitabine, and cisplatin, the median overall survival was 13 months from the initial TACE without any statistical differences between the regimens; in conclusion, TACE is a safe and palliative option in patients with the unresectable disease [57].

Child-Pugh B, hypovascular, and initially progressive disease are poor prognostic factors for patient survival [58,59,60]. Regarding TACE, a prospective trial with nine patients who underwent 30 TACE procedures combined with chemotherapy versus 11 patients treated with chemotherapy alone demonstrated that the overall survival was significantly better in the first group [61]. TACE plus chemotherapy appears to be safe and feasible with encouraging results.

**Figure 8:**
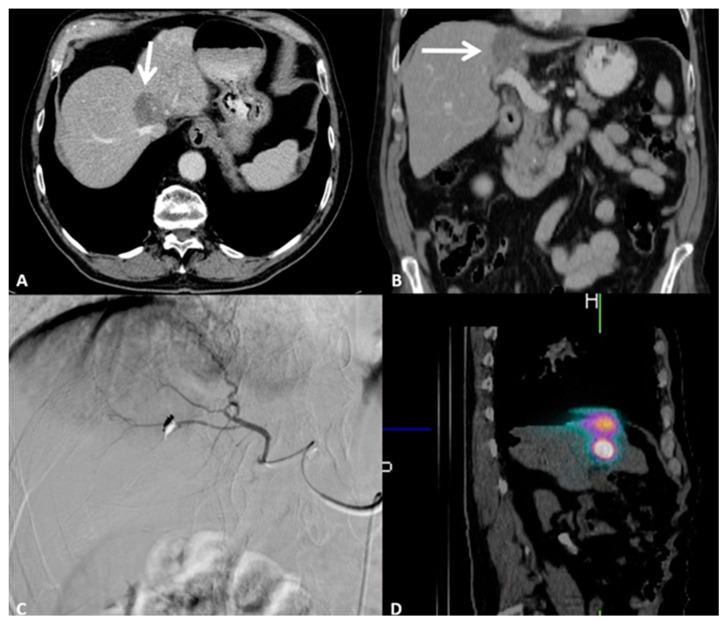
79-year-old male patient presenting with pain in the right upper abdominal quadrant due to intrahepatic cholangiocellular carcinoma. Performance Status One, no surgical indication; the patient underwent eight cycles of chemotherapy. (**A**) Contrast-enhanced axial CT scan showing a 3.5 cm lesion of the I-IV segment adjacent to the inferior vena cava and to the right hepatic vein. (**B**) Contrast-enhanced coronal CT scan showing how the lesion is adjacent to the portal vein. (**C**) Digital subtraction angiography runs showing super selective catheterization of the target vessel with intra-arterial administration of marked albumin macroaggregates, which serve as the diagnostic phase of radioembolization. (**D**) Single-photon emission computed tomography oblique reconstruction scan showing focal and selective uptake of marked albumin macroaggregates by the target lesion, confirming the catheter position for the therapeutic radioembolization.

Regarding transarterial radioembolization (TARE), a retrospective study of 19 chemorefractory patients who underwent TARE has shown that the median survival from diagnosis after the first TARE is about 11.5 months [62]. One more study considered 40 patients undergoing TACE versus TARE in order to understand which treatment to choose in patients with unresectable disease: 25 patients underwent TARE (39 treatments) and 15 underwent TACE (35 treatments) with similar demographics characteristics [63]. The results were similar in terms of overall response rate (TARE 4%, TACE 6%; *p*-value > 0.9), local control rate (TARE 48%, TACE 46%; *p*-value > 0.9), and adverse events (TARE 26%, TACE 20%; *p*-value > 0.9). Recently, a systematic review and meta-analysis were performed to compare TACE and TARE effects for intrahepatic CCA: The primary endpoint was overall survival, and the second endpoint was the clinical adverse effect and tumor overall response [64]. A total of 31 articles were included (1695 patients): 13 were on TACE and 18 were on TARE. Despite clinical and tumor characteristics heterogeneity, the results showed similar median survival and radiological objective response even though TARE showed a better adverse event profile (43% versus 58.5%). The decision on which modality to choose should be based on the experience of the operator and patient-specific factors, such as the liver functionality and lesion dimension (Figure 8) [65].

Individualized dosimetry is mandatory for treatment planning and response prediction in CCA patients undergoing TARE. For a precise evaluation of the activity distribution and dose delivery, dose-response and toxicity investigations, and the therapeutic treatment of extrahepatic deposits, verification imaging studies after TARE are crucial. The majority of research on the use of SIRT in CCA makes use of treatment planning that delivers a mean absorbed dose of 120 Gy to the tumor and a threshold dose of no more than 50–70 Gy to the surrounding healthy liver parenchyma [66]. Various studies have shown sizable disparities between dosimetry verification studies performed after yttrium-90 TARE and the pretreatment predictive planning marked albumin macroaggregates: These differences may result from variations in the flow dynamics, catheter position, and particle size, weight, and density [67,68,69].

### 5.2. Key Points

Locoregional therapies are safe and effective in the treatment of unresectable intrahepatic CCA; these procedures offer a valid alternative for those patients who are ineligible for surgery and provide local tumor control in patients with recurrence after resection. Locoregional therapies obtain better results in the case of single and small (<3 cm) lesions. To date, there is no significant difference between RFA, MWA, TACE, and TARE in therapeutic efficacy; however, data are still limited, and further research is needed.

## 6. Lymphatics and Lymph Node Metastases

The major function of the lymphatic system is to allow the return of plasma proteins from extracellular space into the lumen of capillaries through the wall of endothelial cells [70]. Unfortunately, this pathway represents an easy way for tumors to spread into the organism [71].

The most important component of the lymph node is the stroma, formed by the cortex and the medulla. The cortex can be divided into the outer cortex (containing B-cells) and the paracortex (containing T-cells). The medulla consists of B-cells and macrophages. The lymph node produces new B- and T- cells [72,73].

### Locoregional Approaches

The standard imaging method for the lymphatic system was represented by pedal lymphangiography; however, this procedure is technically challenging and requires the infusion of oily contrast medium over a period of hours [74]. In 1967, Hall and Krementz proposed intranodal lymphangiography (INL), but this procedure was not generalized until 2011 when Nadolski and Itkin demonstrated the feasibility of US-guided INL [75,76].

**Figure 9:**
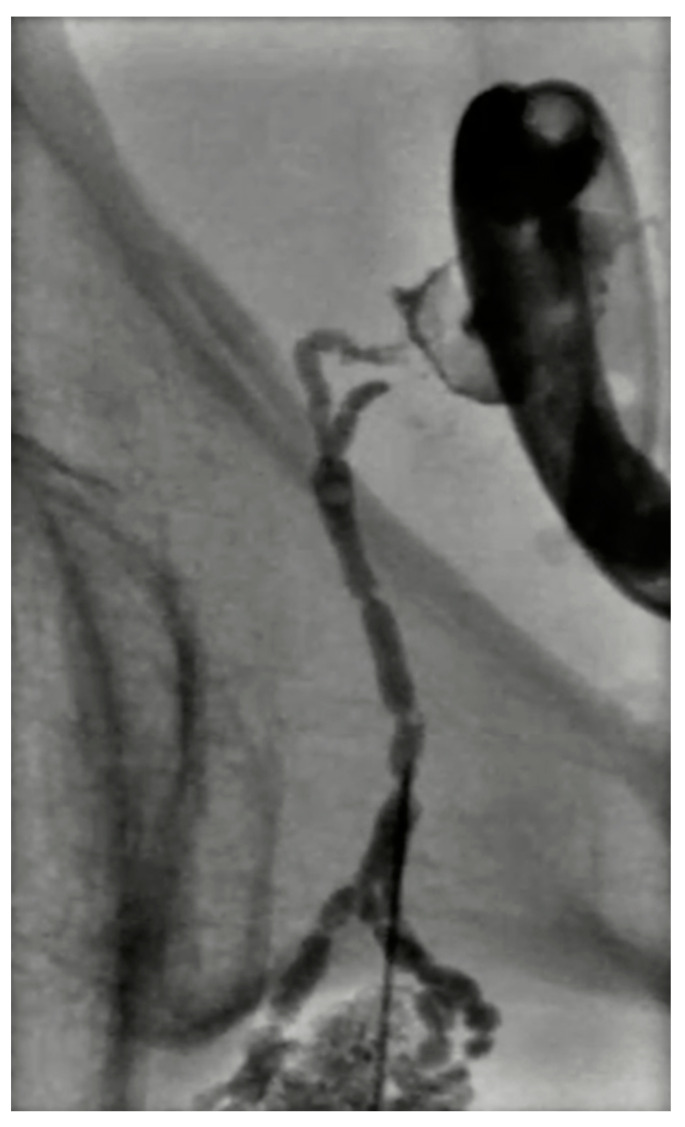
Digital subtraction lymphangiography of a 54-year-old male with lymphocele after prostatectomy. A percutaneous drainage catheter was placed in the lymphocele; then a right obturatory lymphangiography and lymphatic embolization with cyanoacrylate were performed. The percutaneous drainage catheter was removed 2 days later.

The main role of INL is the detection of lymphatic leaks (75% of lymphangiographies with a median fluoroscopy time of 60 min); however, it was soon clear that this technique could be often therapeutic, especially for the treatment of chylous effusions, due to the viscosity of Lipiodol [77,78]. A low effusion volume (<200 mL/day) predicts 97% success, and <500 mL/day predicts 70% success; generally, <1000 mL/day of volume effusion is a good predictor of clinical success. In the last five years, the importance of this procedure in relation to complications after pelvic or retroperitoneal surgery, such as lymphoceles (Figure 9), has been growing [79,80].

Lymph node metastases are very important factors associated with cancer mortality; more specifically, the involvement of lymph nodes represents a predictor of poor prognosis. Current healing strategies for lymph node metastases are represented by chemotherapy, radiotherapy, and lymphadenectomy, but they are burdened by invasiveness, lack of selectivity, and, in the case of chemotherapy, limited access to lymph nodes. For these reasons, there is increased interest in intranodal therapies. The percutaneous administration of immunotherapy or chemotherapy in tumor-draining lymph nodes (regional lymph nodes are typically accessible under US guidance) has shown to be a promising technique [81,82,83,84].

On the other hand, in addition to the abovementioned therapeutic strategies, in recent years, some centers have begun to perform different ablative techniques targeting lymph node metastases. Ding et al. performed a metanalysis to assess the effectiveness and feasibility of thermal ablation, including RFA, MWA, and laser ablation (LA), for lymph node metastases from papillary thyroid carcinoma with interesting results: The total complication rate was 5% with 0% major complications. LA showed a lower complete disappearance rate than RFA whereas there were no significant differences between RFA and MWA in subgroup analysis [85]. Tao Pan et al. evaluated 6-month and 1-year overall survival rates in patients who underwent percutaneous CT-guided RFA of lymph node metastases from HCC, obtaining a 6-month and 1-year overall survival of 87.0% and 62.4%, respectively. Short-term stomach discomfort and a self-limited local hematoma were the only RFA side effects that occurred in a few patients [86]. These studies encourage the use of percutaneous ablation techniques on lymph node metastases, but the relative lack of data does not allow these methods to be introduced into routine clinical practice.

## 7. Bone Metastases

Ablation of bone metastases is indicated for pain palliation, local disease control, and fracture prevention. In case of palliation, it is recommended to consolidate osteolytic metastases with cement or through the use of screws. In the last few years, different studies have registered a growing use of ablation for oligometastatic diseases to the bone, in particular, for radiation-resistant lesions [87,88,89].

### 7.1. Locoregional Approaches

There are three principal indications to treat bone metastasis: moderate-severe pain (>4/10 on the VAS scale) focally localized, easily achievable lesion location, and prevention of complications (i.e., fractures). Up to 3–5 lesions can be treated in a single ablation session [90]. It is not possible to treat spine lesions associated with acute cord compression, widespread metastases, and lesions with active infection [91]. Lesions close to vital structures (arteries and nervous plexuses) should be treated carefully.

Bone metastases are often large lesions situated in weight-bearing locations (spine and pelvic bones). Moreover, bone metastases are frequently localized in areas that had previously undergone radiotherapy, so the bone structure is very unstable and at risk of fractures [92,93].

Cryoablation is the preferred ablative technique in the case of lesions involving the anterior part of the vertebral body whereas RFA is mostly performed in case of posteriorly located metastases [94,95].

**Figure 10:**
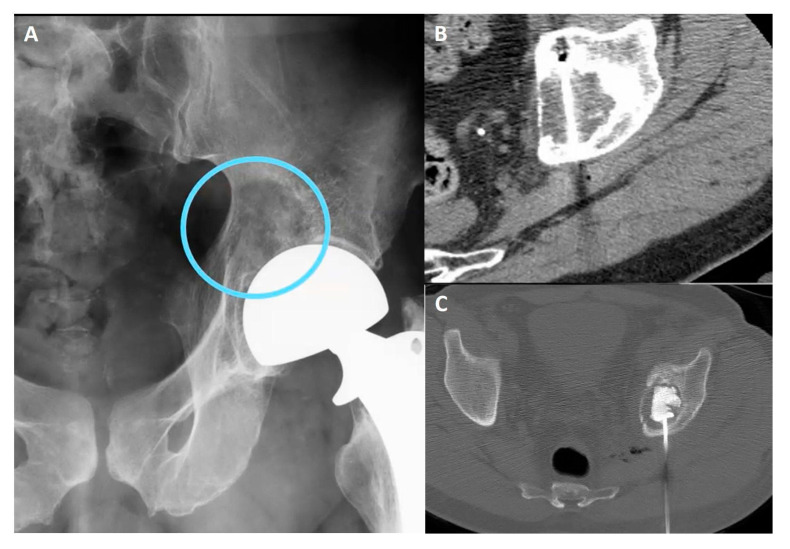
(**A**) Pelvic X-ray showing osteolytic lesion of left periacetabular region (circle). (**B**) Axial CT scan showing cryoablation needle inserted in the osteolytic lesion. (**C**) Axial CT scan showing postcryoablation cementoplasty performed to augment the ablative results and to prevent bone fractures.

Cementoplasty is often performed along the ablation to augment the effects and prevent fractures (Figure 10). It is important to keep in mind the risks of cementoplasty, such as the embolization of cementing material, perineural leak, or intra-articular extension. Consolidation with cement is not always required, as in non-weight-bearing lesions and sclerotic ones [96].

Spine lesions are often burdened by vertebral body collapse; therefore, to decide when cementoplasty is required or not, it is useful to consider the spine instability neoplastic score (SINS), which is based on various characteristics of vertebral metastases (e.g., location, pain, type of bone lesion (lytic, blastic, both)): a score higher than 7 points warrants surgical consultation to assess for instability prior to proceed with any ablative treatment [97].

In addition to cementoplasty, in locations where it is difficult to inject the cementing material, very good consolidation has been obtained using screws [98].

### 7.2. Key Points

The most important result of bone metastases treatment is pain reduction. Several studies have shown pain relief in 75–100% of patients with a pain score relief of approximately 5 points. The rate of complication was reasonably low [99,100]. The most common reported complication is postprocedural pain, typically for the initial 24-h in 70% of RFA, and it is generally managed with therapeutic lines, including nonopioids, opioid drugs, and steroids [101]. Another important treatment result is local tumor control: This is achieved in 85–95% of patients after the procedure both in the case of cryoablation and RFA [102,103]. Different studies have demonstrated the increasingly important role of local bone ablation, especially in the case of oligometastatic disease from renal cell carcinoma [104].

## 8. Conclusions

Interventional oncology is playing a pivotal emerging role in the management of many types of cancer patients. Treatments need to be both effective and safe with a mandatory aim to reduce the risk of complications, offering new potential treatment options with curative or palliative aims. Factors that contribute to this aim include an adequate multidisciplinary approach, correct indication, accurate treatment planning, and comprehensive knowledge of recent technological developments as well as clinical factors affecting treatment efficacy [105].
